# Delay-Dependent Response in Weakly Electric Fish under Closed-Loop Pulse Stimulation

**DOI:** 10.1371/journal.pone.0141007

**Published:** 2015-10-16

**Authors:** Caroline Garcia Forlim, Reynaldo Daniel Pinto, Pablo Varona, Francisco B. Rodríguez

**Affiliations:** 1 Laboratório Fenômenos Não-Lineares, Instituto de Física, Universidade de São Paulo, São Paulo, Brazil; 2 Lababoratório Neurodinâmica/Neurobiofísica, Instituto de Física de São Carlos, Universidade de São Paulo, São Carlos, Brazil; 3 Grupo de Neurocomputación Biológica, Departamento de Ingeniería Informática, Escuela Politécnica superior, Universidad Autónoma de Madrid, Madrid, Spain; Universität Bielefeld, GERMANY

## Abstract

In this paper, we apply a real time activity-dependent protocol to study how freely swimming weakly electric fish produce and process the timing of their own electric signals. Specifically, we address this study in the elephant fish, *Gnathonemus petersii*, an animal that uses weak discharges to locate obstacles or food while navigating, as well as for electro-communication with conspecifics. To investigate how the inter pulse intervals vary in response to external stimuli, we compare the response to a simple closed-loop stimulation protocol and the signals generated without electrical stimulation. The activity-dependent stimulation protocol explores different stimulus delivery delays relative to the fish’s own electric discharges. We show that there is a critical time delay in this closed-loop interaction, as the largest changes in inter pulse intervals occur when the stimulation delay is below 100 ms. We also discuss the implications of these findings in the context of information processing in weakly electric fish.

## Introduction

Weakly electric fish produce and perceive electric signals. Pulse-type electric fish have electroreceptors across their bodies and an organ that generates all-or-nothing species-specific electric organ discharges (EODs) [1–5]. EODs are used to sense the environment, search for food, identify sex and species and to communicate. EODs can be easily detected non-invasively, thus, weakly electric fish are a convenient animal model to study information processing and communication mechanisms in freely behaving animals. The EOD waveform is stereotyped among discharges, on the other hand, the interval between theses discharges, known as the inter pulse intervals (IPI), varies considerably (from ~10–400 ms) and carries information about the behavioral state of the fish [6–8]. Depending on the behavioral context (e.g. night/day) and the external stimuli, fish adapt their electric behavior [6,8–10] by changing their IPI distribution over time. These changes in the temporal structure of the EODs are also correlated with different swimming patterns and overall motor behavior [9,11,12].

*Gnathonemus petersii* and other Mormyridae exhibit a well studied echo response. They fire a single pulse approximately 12 ms after receiving another external electrical pulse [1,13–16]. The echo response can be observed for a stimulus sent not further than 30 cm away from the fish [16]. This mechanism could avoid electrical jamming between the EODs of different fish [12,14] and might also be involved in communication [13].

Many neuroethological experiments in weakly electric fish have used playback recordings to stimulate the animals [6,7,12,17–21]. Playback stimuli consist of pre-recorded signals that are delivered independently of the fish's response. Weakly electric fish respond differently to stimuli recorded from attacking fish, resting fish and randomized sequences of IPIs [7,12,17]. The precise timing of the pulses generated by the fish, presumably arising from the interaction with the environment, may play an important role in the information coding mechanisms.

Although the importance of playback experiments is unquestionable, the history-dependent nonlinear and adaptive nature of the nervous system makes advisable the use of other types of stimulation protocols that can take into account the actual (neural and behavioral) state of the animal. The ability to change a stimulus as a function of real time events detected in the ongoing activity can expose relevant aspects hidden under traditional open-loop stimulation protocols and bridge between different levels of analysis.

Activity-dependent stimulation techniques in Neuroscience have been widely applied under the dynamic clamp concept for electrophysiological experiments “in vivo” and “in vitro”. In recent years, closed-loop protocols have been used in many different description levels ranging from single neuron and motion controlled sensors to behavioral experiments in animals [10,14,16,22–28], and EEG, ECoG and fMRI studies in humans [29–31]. There is a wide variety of possibilities to monitor animal behavior and deliver activity-dependent stimuli which can have an adaptive temporal structure based on events detected from signals or behavior produced by the animals. For electric fish, these events can be monitored from the fish's own electric activity [22]. The electric activity consists of short pulses, ~1 ms long, which have to be detected in real time in order to implement the activity-dependent stimulation protocol.

Closed-loop experiments were first applied to *Gnathonemus niger* in the 70's where the variation of EOD frequency elicited by controlled electric stimuli was analyzed in confined fish [10]. In *Gnathonemus petersii*, this technique was mostly used to study the echo response [14,16]. In both species the stimulus consisted of square pulses delivered to confined fish [18].

In this paper, we have developed a simple protocol to study the response of freely swimming *Gnathonemus petersii* to naturalist waveform pulse stimulation delivered each time the fish produces an EOD. We show that differences in the relative timing of the fish EOD and the external stimulus, referred as the stimulus delay, affected the fish response altering the IPI probability distribution. In particular, we analyzed the effect of different stimulus delays in the closed-loop stimulation as compared to the spontaneous electrical activity of the fish. We observed that exists a critical time delay of 100 ms. Under stimulus delays shorter than this value, frequency for all ranges of IPIs increased, on the other hand, delays larger than 100ms, induced a variety of behaviors in different IPI ranges.

## Methods

### Animals

Specimens (N = 5), 5–12cm long, of *Gnathonemus petersii* were acquired from local dealers (Aquarium de Madrid, C/ Maestro Victoria, 8) in Madrid, Spain. No information about sex was available. Fish were housed in a 30 L (40 x 30 x 25) cm tank, water temperature was kept at 25°C, exposed to natural illumination (10:14 light:dark cycle) and provided with PVC pipes as hiding places. Water conductivity was 124 μS/cm and pH = 7.3. Fish were fed once a day with *Artemia salina* (brine shrimp) or bloodworms.

Permission of the ethics committee of Universidad Autónoma de Madrid was obtained (TIN2012-30883). All experiments were noninvasive behavioral trials: fish were stimulated using weak electric pulses with the same amplitude as their natural electrical activity. All animals behaved normally after the experiments.

### Measurement aquarium

**Fig 1:**
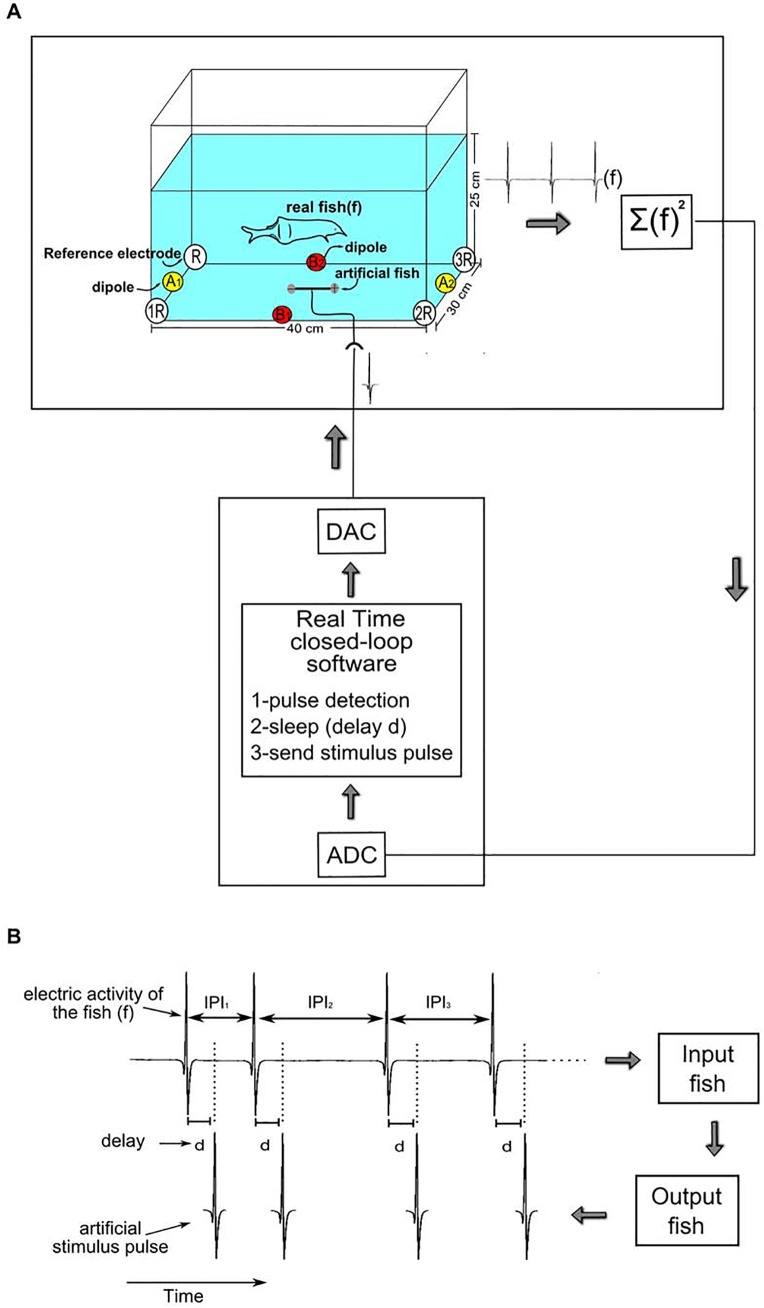
Setup for closed-loop activity-dependent protocol. A–Experimental Setup. The EODs were measured using 8 electrodes, placed on the bottom of the tank (40×30×25) cm. The electrodes were connected to form an array of 5 dipoles: R-1R, R-2R and R-3R, sharing a common reference (R; white circles), A1-A2 (yellow circles) and B1-B2 (red circles). The signal from the 5 dipoles were differently amplified (gain = 50x or 100x: for small fish ~5 cm), summed, squared and then digitized at 25kHz by an ADC board (NI PCI-6521) and stored for posterior analysis. The stimulus pulses were generated by the same ADC board and controlled in real time by a closed-loop real time software that detected the EODs timing and delivered stimulus pulses in response. The stimulus pulses were delivered to the tank by a 7 cm dipole (artificial fish) to mimic an average size of *Gnathonemus petersii* used in this study. The artificial fish was placed in the middle of the tank as shown in the figure. B–Closed-loop activity-dependent protocol. The real time closed-loop software detected the EODs timing and sent a stimulus pulse after a time delay (*d)* chosen by the experimenter. The stimulus pulses had the exact *Gnathonemus petersii* waveform and 3V amplitude to mimic an average size animal. All experiments were performed with the same amplitude and wave shape.

The experiments were performed in a (40 x 30 x 25) cm tank (30 L) with water temperature at 25°C. To measure the fish's EODs (Fig 1A), 8 silver tip electrodes were placed on the bottom of the tank, 4 at the corners and 4 at half distance (Fig 1A–colored circles), and connected to form an array of 5 dipoles: R-1R, R-2R and R-3R, sharing a common reference (R; white circles), A1-A2 (yellow circles) and B1-B2 (red circles; as shown in Fig 1A). Signals from the electrodes were differentially amplified (TL082, Texas Instruments; gain = 50x or 100x: for small fish ~5 cm) summed (UA 741, Texas Instruments) and squared (AD633, Texas Instruments). The squared signal was digitized at 25kHz by an ADC board (NI PCI-6521, National Instruments Corporation). Such configuration, adapted from [9] and originally designed to measure the activity of *Gymnotus carapo*, allows fish of all sizes to have their EODs detected while freely swimming due to an optimal cubic configuration of the electrodes resulting in 7 dipoles and to the fact that the signals measured in each dipole is squared and all 7 squared signals are summed. The setup was adapted to *Gnathonemus petersii* size and motor activity. *Gnathonemus petersii* remain most of the time swimming close to the bottom of the tank, so all electrodes were placed at the bottom. Two dipoles were also placed in the middle of the glass walls (B1-B2 and C1-C2 in Fig 1A) instead of in the corners. This configuration reduced the 7 dipole (cubic configuration) to a simpler 5 dipole one but yet keeping its main features. The total number of dipoles in the tank must be chosen as a compromise between the size of the animal and the size of the tank.

To deliver stimulus pulses, a 7 cm silver tip dipole was placed on the bottom in the middle of the tank (Fig 1A). The stimuli were generated by a quad-core computer and delivered by the same DAC board used for recording the activity (NI PCI-6521, National Instruments Corporation). The computer run the RTBiomanager real-time closed-loop software to build the activity-dependent stimulation [22,23,32–34]. The stimulus pulses had the *Gnathonemus petersii* characteristic waveform and 2.4 V of amplitude in total. The EOD waveform had been previously recorded with a dipole from a 7 cm fish inside a small plastic recipient, so fish could not move altering the pulse amplitude, and stored in the computer. The same EOD waveform and 2.4 V amplitude were used in all experiments.

To study how precise stimulus timing affects the fish's IPI distribution, we developed a closed-loop protocol consisting of monitoring the electric pulses of the fish and delivering stimulus pulses at different time delays *d* (Fig 1B). The precise detection of the EOD is crucial in order to stimulate fish according to this activity-dependent protocol. In our setup, this precise detection was implemented by the real time closed-loop software which also precisely delivered the stimulus pulses after the time delay specified by the experimenter (Fig 1B). The squared signal recorded from the fish and the stimulus signal sent to tank were both stored for further pulse time offline analyses. During the closed-loop stimulation sessions, the signal recorded from the tank contained both fish and stimulation pulses. However, for the analysis of IPI distributions only the IPIs from the live fish were used. Thus, to isolate the pulses that were discharged by the fish, we synchronized the stimulus file and the file with the tank recording signals and removed the pulses that appeared in both files.

To assess how fish react to a stimulation signal that depends on the timing of the EODs produced by the fish, we developed the following closed-loop protocol: a 30 min control session to record fish's spontaneous activity without any stimulation followed by a 30 min closed-loop stimulation session. Fish were subjected to this protocol from 2 to 4 times a day. During the closed-loop stimulation session, when the fish discharged a pulse, a stimulus pulse was delivered to the fish after a fixed time delay *d* for 30 min (Fig 1B). The delays chosen were in the range of *Gnathonemus petersii* IPIs: 5, 10, 12, 22, 32, 40, 45, 50, 60, 70, 100, 102, 162, 172, 202, 282 ms. We performed, in total, 22 experiments at different times of the day with 5 different fish (Table 1 lists the complete stimulus schedule. One fish was used each day. The fish was placed in the measurement aquarium one night prior to stimulus presentation. Time delays were randomly chosen for each fish so that all fish were stimulated by different delay ranges.

**Table 1:** Corresponding fish and delays used in this study.

Delay (ms)	Fish A	Fish B	Fish C	Fish D	Fish E
5				X	
10				X	
12			X		
22	X				
32		X			
40				X	
45	X				
52					X
60				X	
70				X	
100				X	
102	X	X	X		X
162			X		
172		X			
202	X				
282			X		

For each session we computed the IPI histograms, plotted the quantile-quantile, calculated the area under the quantile-quantile curves and the Tukey mean-difference (IPIs from the stimulus session–IPIs from the control session) [34]. All data are available at http://www.ii.uam.es/~gnb/material.htm.

In quantile-quantile plots, the data (IPIs) from 2 distributions X and Y are sorted and plotted against each other X vs Y, the black line represents the reference line y = x, slope = 1. If IPIs from the control sessions (X) and the stimulus sessions (Y) come from the same IPI distribution, the qqplot will show points lying on or parallel to the reference line, indicating that there were no changes in the electrical activity due to the experiments:

- if points lie exactly on the reference line, it means that the closed-loop stimuli do not affect the fish,
- if the points lie on a line of slope = 1 above the reference line, the IPIs discharged during the stimulus session are longer than those of the stimulus session and both come from similar IPI distributions, if the points lie below the reference line it means that IPIs discharged during the stimulus session are shorter than those of the control session,
- when points lie on a line with slope different than 1, the points that remain above the reference line are those in which the IPIs from the stimulus session are longer, and the ones below the reference line are those in which the IPIs discharged during the stimulus session are shorter.

## Results

**Fig 2:**
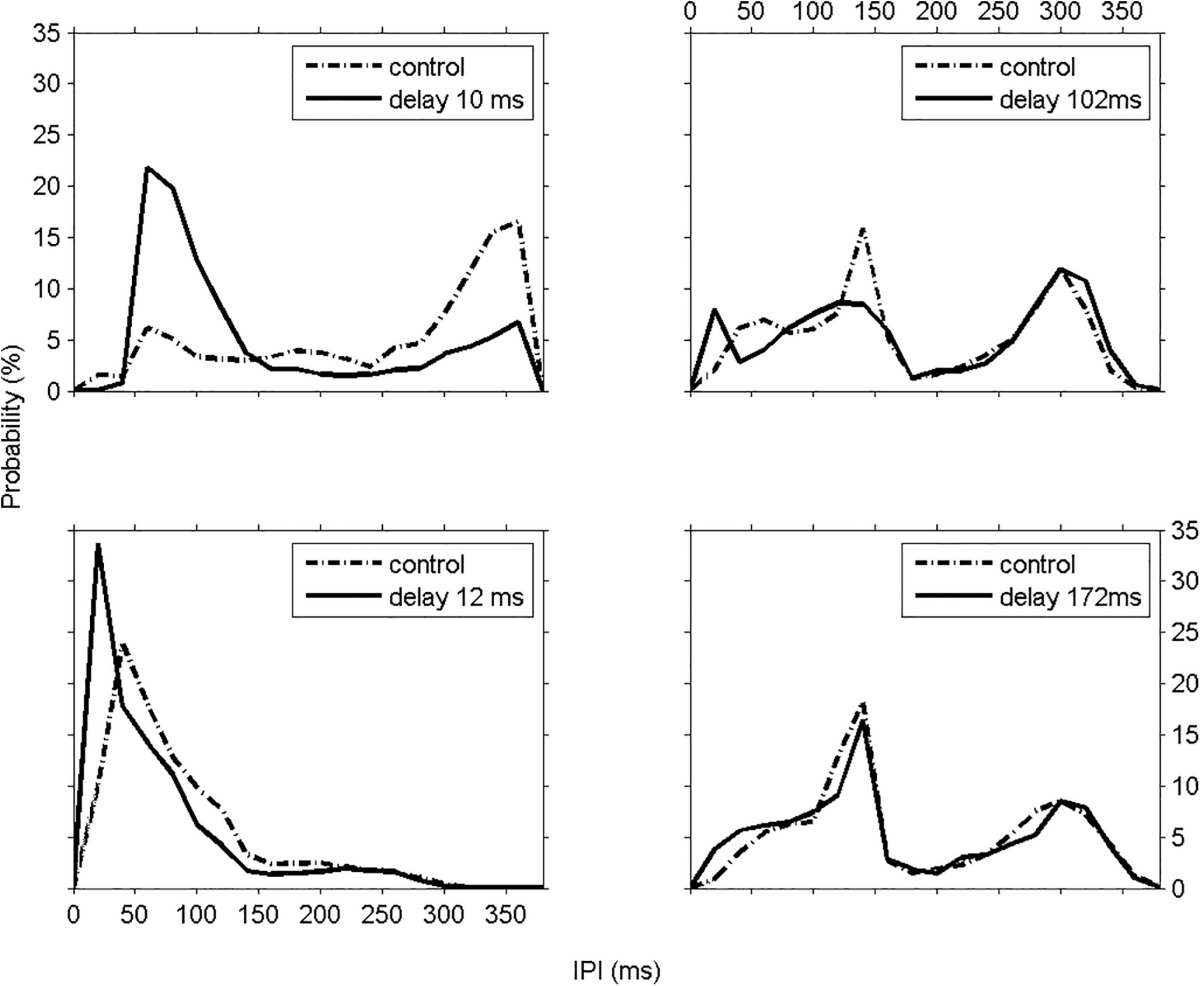
Fish response depends on the closed-loop stimulus time delay. IPI distributions of 4 different experiments are shown for control (dashed lines) and closed-loop stimulation sessions (solid lines), each panel represents the data of a single fish subjected to a single delay for 30 min, the IPIs used to build the histograms were defined in Fig 1B. Top-left–For the stimulus session with time delay *d* = 10 ms (full line) fish increased the discharges of shorter IPIs, specially around 60 ms IPIs and decreased the probability of firing longer IPIs (> 300 ms) when compared to those of the control session (dashed line). There were also changes in the overall shape of the IPI distribution (50% Pearson's corr., Kolmogorov-Smirnov (KS) p = 0.004). Bottom-left–When stimulated with pulses with time delay *d* = 12 ms (solid line), fish shortened its IPIs from 5 ms to 200 ms, that is, there was an increase in the frequency of the electric organ as compared to those for the control session (dashed line). For longer IPIs (>200ms) no changes were observed between control and stimulus sessions, i.e., the shape of the IPI distribution remained the same in both sessions with a shift of ~20 ms (52% Pearson's corr., KS p = 0.07). Top-right–For the time delay *d* = 102 ms (full line), fish increased the probability of firing shorter IPIs of ~20 ms and also longer IPIs (> 300 ms), and decreased the probability of discharging IPIs around 140 ms as compared to those of the control session (dashed line). There were slight changes in the shape of the IPI distribution (89% Pearson's corr. KS p = 0.8). Bottom-right—For stimulus session with time delay *d* = 172 ms (solid line), fish discharged with high probability 140 ms IPIs and 300 ms IPIs and the shape of the IPI distribution did not change (97% Pearson's corr. KS p = 0.4).

Fish reacted differently, increasing/decreasing their IPIs depending on the delay *d* used to deliver the stimulus. In Fig 2 we show representative examples of 4 experiments where one can observe different IPI responses to different delays as compared to the control sessions: increase of frequency (shorter IPIs) and change in the IPI distribution shape (50% Pearson's corr., kolmogorov-smirnov (KS) p = 0.004; 10 ms delay; Fig 2 –top left), increase in frequency, but no changes in the shape of IPI distribution (52% Pearson's corr., KS p = 0.07; 12 ms delay; Fig 2 –bottom left), slight decrease/increase of frequency changing the shape of distribution (89% Pearson's corr. KS p = 0.8; 102 ms delay; Fig 2 –top right) and slight changes in frequency and similar IPI distributions (97% Pearson's corr. KS p = 0.4; 172 ms delay; Fig 2 –bottom right). Each panel represents the data from a single fish subjected to a single delay for 30 min, the IPIs are shown in Fig 1B.

In the experiment with delay 10 ms we observed that the fish changed the IPI distribution firing with high probability (from 6% to 22%) of shorter IPIs around 60 ms (Fig 2 –top left–solid line) as compared to the control session that presented high (16.5%) probability of long IPIs around 360 ms (Fig 2 –top left–dashed line). The stimulus session with delay 12 ms (Fig 2 –bottom left–solid line), the IPI distribution was shifted 20 ms to the left in the range from 0 to 200ms, with high probability (from 10% to 34%) of 20 ms IPIs and remaining the same (~2%) from 200 ms to 300 ms. For the 102 ms delay (Fig 2 –top right–solid line), the firing probability of short IPIs around 20 ms increased (from 2% to 8%) and decreased for IPIs around 140 ms (from 16% to 8.5%) and IPIs longer than 300 ms. For the 172 ms delay (Fig 2 –bottom right), the shape of the IPI distribution for the control session (dashed line) and stimulus session (full line) were not significantly different (KS p = 0.4, 97% pearson's corr) with high probability (16.5%) of 140 ms IPIs and 300 ms IPIs (8.5%). In this case, there was also an increase in the probability (from ~2% to 4%) of discharging shorter IPIs from 0 to 100 ms during the stimulus session (solid line).

**Fig 3:**
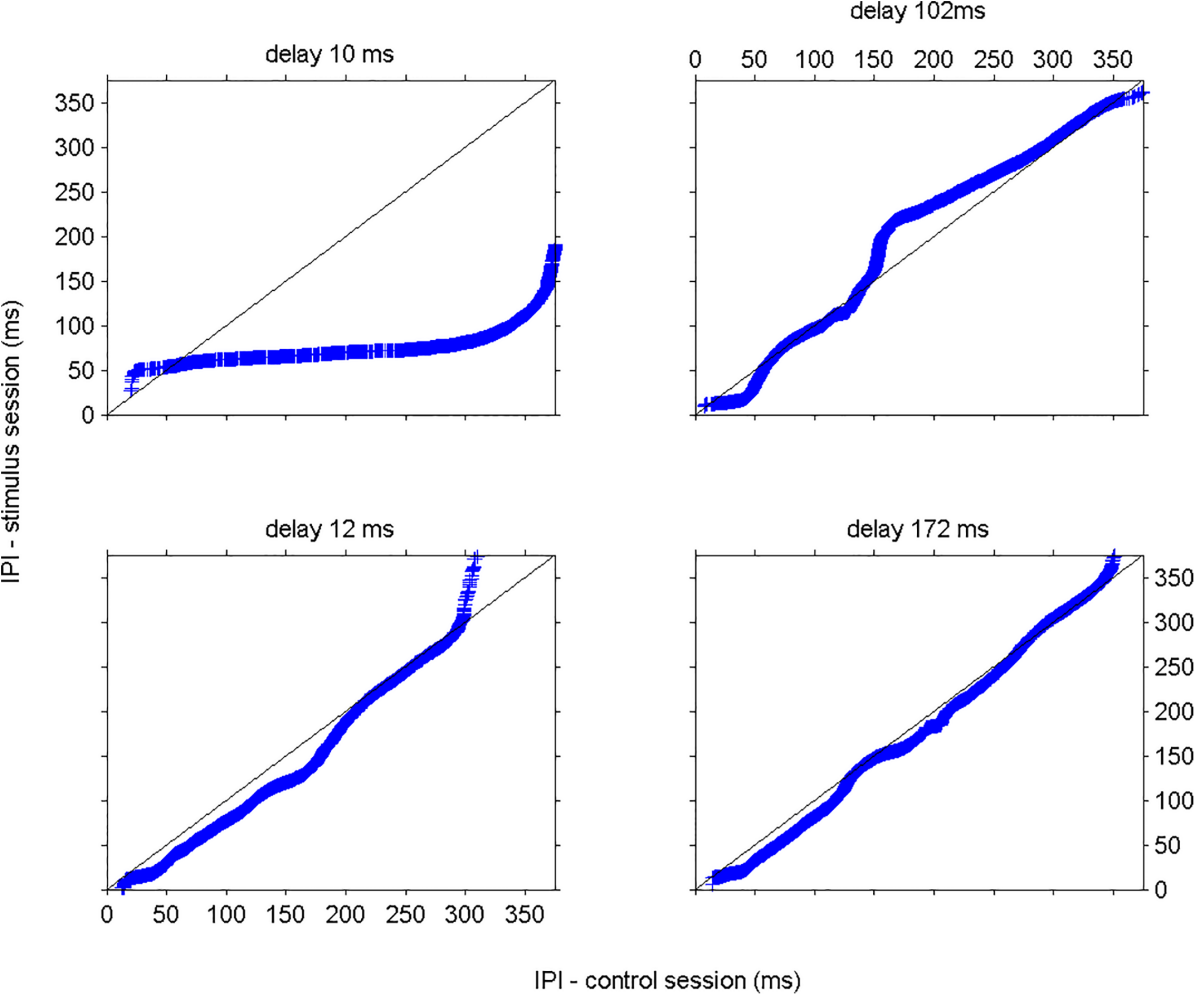
IPI changes depend on the stimulus time delay. Quantile-quantile plot of IPIs during control and the closed-loop stimulation sessions for 4 different experiments. The black line represents the reference line y = x, slope = 1. Top-left–the IPIs from the control session and stimulus session with time delay d = 10 ms came from different distributions. Most of the points (blue) were under the reference line indicating that IPIs discharged during the stimulus session were shorter than those of the control session in the range from 50 ms to 375 ms. Bottom-left–The IPIs discharged during the 2 sessions come from similar distribution, IPIs discharged during the stimulus session were ~20ms shorter in a range from 15 to 200 ms. Top-right–IPIs ranging 60 ms to 150 ms came from similar distributions in the control session and in the closed-loop stimulation session. For longer IPIs (>150 ms), the control session presented always shorter IPIs than those of the closed-loop stimulation session, the opposite happened to IPIs shorter than 60 ms. Bottom-right–IPIs from both sessions came from shifted distributions.

To quantify the changes in the IPI distributions between control and stimulus sessions, we show the quantile-quantile plots (qqplot) [35], in Fig 3, a well-known statistics tool to measure differences in distributions from 2 datasets in Fig 3, for 4 different experiments (presented in Fig 2). If the qqplot curve show points that are not forming a straight line, the IPIs from the stimulus sessions and control sessions come from different IPI distributions, that is, the closed-loop stimulation session largely affected the fish.

When stimulated with delay 172 ms (Fig 3 –bottom right), the IPIs from control and stimulation sessions were similar (point lying around the reference line), indicating that this particular delay did not affect the fish, as can be seen in Fig 2 –bottom right. The IPIs discharged during the control session and stimulus session with 10 ms delay (Fig 3 –top left) were different, moreover, most of the points (blue) lied under the reference line (black) indicating that the IPIs from the stimulus session were shorter than those of the control session in the range of 50 ms to 375 ms, as also observed in Fig 2 –top left. For a delay of 12 ms (Fig 3 –bottom left), one can observe that IPIs from the control and the closed-loop stimulation sessions came from similar distributions (points formed a straight line) from 15 ms to 200 ms, although they are shifted about 20 ms, that is, fish discharged IPIS during the stimulus session that were, in average, 20 ms shorter than those of the control session; in Fig 2 –bottom left–one can see a shift to the left in IPI histogram for the stimulus session. Both distributions were similar (straight line) for IPIs from 200 ms to 300 ms. The control session and stimulus session for delay 102 ms (Fig 3 –top right) presented only similar distribution for IPIs from 60 ms to 150 ms. For IPIs longer than 150 ms and shorter than 60 ms, the distributions were different: the points above the reference line indicate that the fish discharged longer IPIs during the closed-loop stimulation session as compared to the control one, and points under the reference line show that shorter IPIs were fired during the closed-loop stimulation than those during the control session.

**Fig 4:**
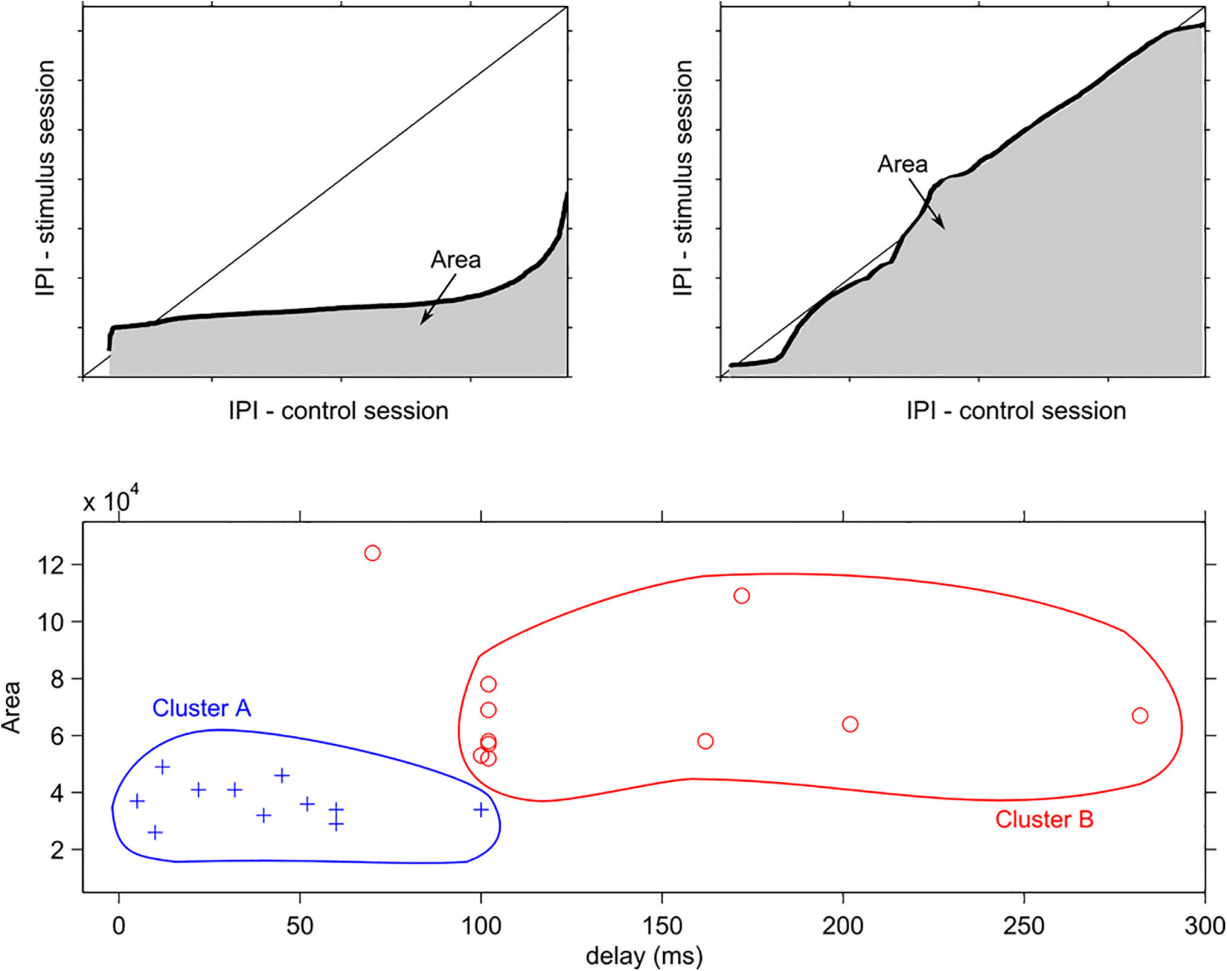
Clustering of IPI changes and analysis as a function of the time delay and the area under the quantile-quantile plot. If the IPIs discharged during the closed-loop stimulation sessions are shorter than those of the control sessions (top-left), the area under the qqplot curve are smaller than that for the case when IPIs from the 2 sessions come from similar IPI distributions (top-right). The area values for all 22 experiments where individually plotted against the time delays (bottom). The area values were clustered in 2 groups separated by a critical delay *d* = 100 ms, cluster A (blue crosses; n = 11) containing time delays from 5 ms to 100 ms (except *d* = 70 ms which was treated as an outlier) and cluster B (red circles; n = 10) from 100 ms to 280 ms. The areas in cluster A were smaller than those of cluster B.

For all 22 experiments, we observed that there was an increase in the probability of firing shorter/longer IPIs for small/large delays, that is, fish were reacting differently depending on the delay. To further quantify these changes, we calculated the area under the qqplot curve (Fig 4 –top). If shorter IPIs were discharged and the IPI distributions were different in the closed-loop stimulation sessions as compared to the control ones, the area under the curve would be smaller (Fig 4 –top left) than those for the opposite case: similar IPIs and similar distribution (Fig 4 –top right). Hence, small areas mean that fish were affected, increasing their frequency by the activity-dependent stimuli, and large areas mean that they were less affected.

To assess how the area under the qqplot curves is related to time delays, we plotted area *versus* delay and ran a standard K-means clustering algorithm (Fig 4 –bottom). We found 2 cluster (silhouette coefficient = 0.72 [36]. Smaller area values were concentrated in the first half of the time delays, from 5 ms to 100 ms (the fish subjected to delay *d* = 70 ms was treated as an outlier because the fish was, probably, not healthy and died a few days later) forming the first cluster (n = 11), named A (Fig 4 –bottom–blue). The second cluster (n = 10), named B, was formed by delays larger than 100 ms, with higher area values (Fig 4 –bottom–red).

**Fig 5:**
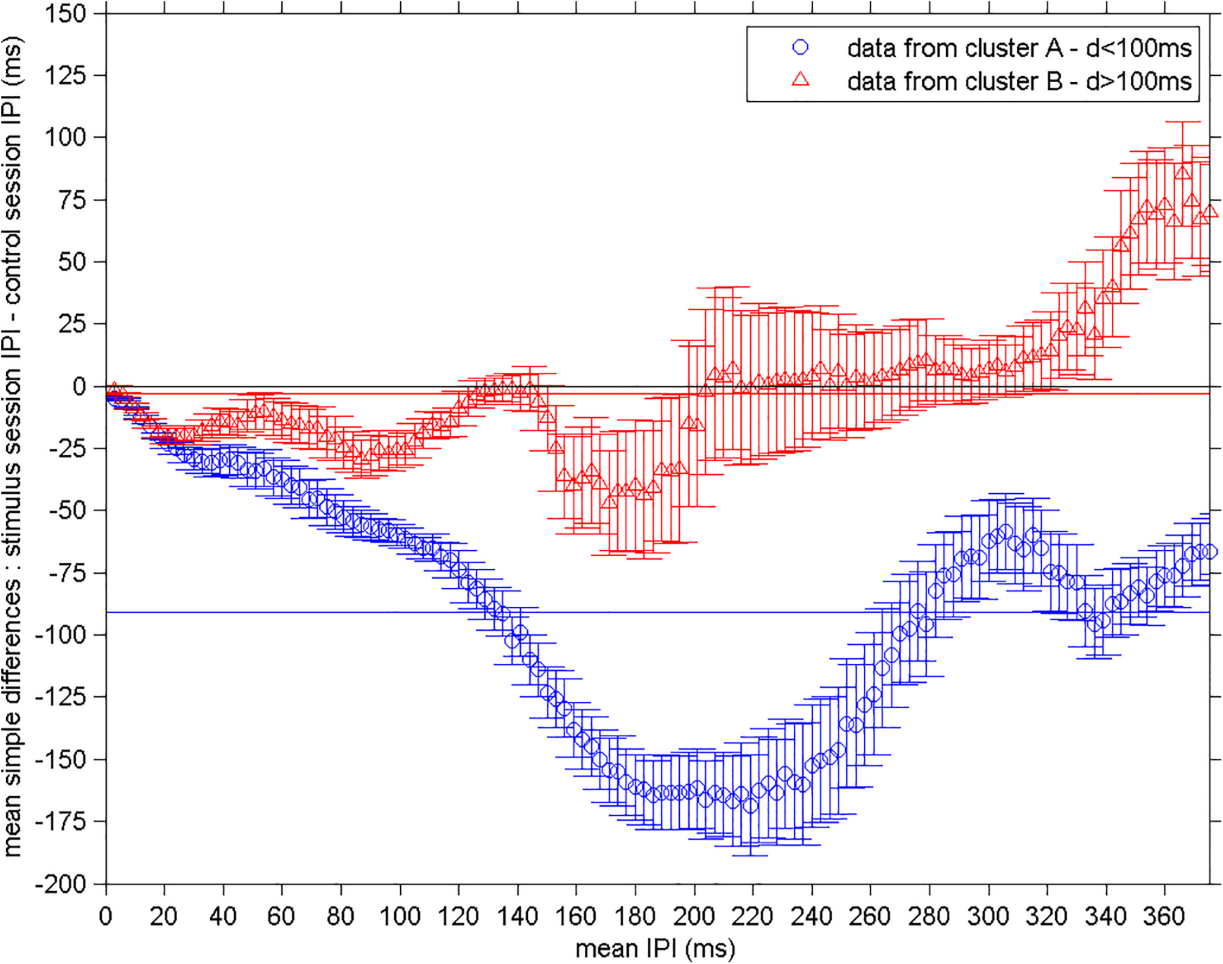
Global IPI changes differences for 2 clusters. Mean simple IPI differences between control (X) and stimulus sessions (Y). For the 11 experiments in each cluster in Fig 4, we calculated individually the Tukey mean-difference (Y-X), took the average integrating them in steps of 3 ms and the mean standard deviation (error bars). For cluster A (blue circles), time delays < 100 ms, IPIs discharged during the stimulus sessions were, in average, 90.8 ms shorter than those of the control sessions (blue line), average IPIs from 187 ms to 235 ms presented maximum differences. For cluster B (red triangles), time delays > 100 ms, IPIs discharged during the stimulus sessions were, in average, 3 ms shorter than those of the control sessions (red line). IPIs from 118 ms to 151 ms and from 199 ms to 319 ms were not altered by delays above 100 ms, although IPIs in the last interval had large deviations from the mean. The maximum change in IPIs happened in a range from 157 ms to 196 ms, where fish fired IPIs ~36 ms shorter in the stimulus sessions compared to those in the control sessions. Fish discharged longer IPIs during the stimulus sessions in the IPI ranges from 322 ms to 376 ms. IPIs up to 25 ms were altered independently of time delay.

To further analyze the impact of the closed-loop stimulation on the behavior of fish's EOD, we divided the 22 experiments into 2 clusters, one with datasets from experiments with delays shorter than 100 ms (cluster A) and other with the datasets from delays longer than 100ms (cluster B). For this analysis we used the Tukey mean-difference (Y-X) [35], which accounts for the simple differences between control session IPIs (X) and the stimulation session ones (Y), and computed the average for each cluster (Fig 5 –cluster A–blue circles and cluster B–red circles) and mean standard deviation (error bars in Fig 5). This figure shows the average changes in the IPIs discharged during the closed-loop stimulation sessions as compared to the IPIs discharged during the control sessions. The Tukey mean-difference [35] is the difference between the 2 data sets (Y-X) and it is calculated for each point in the quantile-quantile plot. If the stimulus does not alter the fish's EODs, the sets X and Y will be very similar and, the Tukey mean-difference values will be close to 0. If the data lie on the zero line, it means that there were no changes in IPIs due to the closed-loop stimuli.

For short IPIs up to 25 ms, the changes in the IPIs did not depend on the stimulus delay, fish discharged equally shorter IPIs when stimulated by delays of both clusters (red and blue circles were superimposed, KS p = 0.9). For IPIs larger than 25 ms, fish were affected depending on the delay (KS p = 0.0001). For cluster A (blue circles; delays < 100ms) all points were negative meaning that for all IPI ranges fish fired shorter IPIs in comparison to the control sessions. IPIs discharged during the closed-loop stimulation sessions were, on average, 90.8 ms shorter than those of the control sessions (blue line); average IPIs from 187 ms to 235 ms presented maximum differences (minimum point in Fig 5 –cluster A–blue circles). For cluster B (red triangles), that is, time delays > 100 ms, IPIs discharged during the stimulation sessions were, in average, 3 ms shorter than those of the control sessions (red line). IPIs from 118 ms to 151 ms were not affected by the closed-loop stimulation using delays of cluster B (red triangles along on the black zero line–cluster B). The biggest changes happened for IPIs from 157 ms to 196 ms, where fish fired IPIs, on average, 36 ms shorter during the stimulation than those of the control sessions (minimum point in Fig 5 –cluster B–red triangles). The firing of IPIs from 199 ms to 319 ms were not, on average, altered by the stimulus with delays in cluster B, although the mean standard deviations were considerably large (red triangles close to the black zero line–cluster B). Fish changed their activity when discharging IPIs longer than 322 ms, by discharging IPIs, on average, 50 ms longer in the stimulus sessions as compared to the control ones.

## Discussion

Weakly electric fish are a convenient animal model to study information coding and communication mechanisms in alive and active fish by interacting with them in a closed-loop fashion. Most studies in the literature take into account that information is encoded in sequences of pulses [6,7,11,12,17]. Stimuli delivered to the fish are typically pre-recorded from other animals, which means that changes in the response of the fish to the stimuli are not considered in these experiments. Closed-loop experiments in weakly electric fish have been mostly used to address navigation [24], jamming avoidance response models [26], in dynamic clamp *in vitro* studies [27] and to characterize the echo response [10,14,16]. The pioneering experiments with echo response were typically studied using artificial EOD timings with square pulses and fish confined in narrow cages.

In this paper we adapted a setup described in Forlim and Pinto (2014) [9] to study the response of a freely swimming *Gnathonemus petersii* under closed-loop stimulation with different stimulus delivery delays *d*. The setup used multiple electrodes placed on the bottom of the tank and realistic stimulus pulses with the characteristic *Gnathonemus petersii* waveform. The setup is very flexible, so it can be easily adapted to other species of wave and pulse-type weakly electric fish. The activity-dependent closed-loop protocol consisted in delivering a stimulus pulse in response to the fish's EODs. We studied the response of the fish, given by the IPI distribution, under this activity dependent protocol as compared to the activity observed without stimulation.

Our results show that information processing in pulse-type weakly electric fish can take into account response delays on a single pulse basis. Depending on the stimulation delay, *Gnathonemus petersii* generate different EOD behaviors as measured by their IPI distributions. This critical delay divided those closed-loop stimulation experiments that affected most of the fish from those that slightly affected them. Affected, here, means an increase in frequency (shortening of the IPIs). For delays shorter than the critical value of 100ms, the probability of firing shorter IPIs increased, i.e., fish increased the frequency of their electric organ. The IPIs were, in average, up to 168.5 ms shorter than those discharged when there were no stimulation (control sessions). The average decrease, for all ranges of IPIs, was 90.8 ms.

When stimulated with time delays longer than the found critical value of 100 ms, fish fired IPIs, in average, 3 ms shorter (see red line in Fig 5) than those of the control sessions. The highest differences were found for IPIs ranging from 157 ms to 196 ms (see minimum points in Fig 5 –cluster B–red triangles); for this range, IPIs from the stimulus sessions were up to 36 ms shorter (see minimum point in Fig 5 –cluster B–red triangles) than those for the control sessions. IPIs from 118 ms to 151 ms and from 199 ms to 319 ms were not altered by the presence of stimulus pulses (they lied closed to the zero black line in Fig 5). IPIs from 322 ms to 376 ms (Fig 5 –cluster B–red triangles) were, in average, 50 ms longer during the stimulus sessions, which means a decrease in the frequency of the electric organ. As a final result, we observed that smaller time delays evoked larger changes in the IPI distributions.

It is worth noting that changes observed from ~8ms to ~20 ms were independent on the delay *d* (Fig 5- blue and red curve presented nearly identical decays). These IPIrange is comparable to those of well-known mechanisms, such as, echo response [13,14,16] and novelty response [1,37]. Echo response has been described to be present if the stimulus pulses are sent 30 cm away from the fish and for pulse delays longer than 10 ms [16]. Novelty response is a transient shortening of IPIs in response to novel external factors [37]. In agonistic encounters, shortening of IPIs has also been observed linked to chasing, attacking, and intense aggression with IPI pattern changes known as smooth acceleration, burst, rasp, and tonic patterns [6,11–13,38].

Our results showed not only changes from 8 ms to ~20 ms but also in longer IPIs. Moreover, the alterations in longer IPIs depend on the stimulus delay and, they cannot be solely explained by the behaviors described above. We hypothesize that alterations in IPIs longer than 20 ms present further evidence that fish encode information on a single pulse. It is tempting to speculate that changes in long IPIs might be involved in communication, for example, fish could tune their pulses to follow within a certain interval after a conspecific's discharge. Tuning of pulses to specific time intervals was already demonstrated in the Southern-American pulse-types *Gymnotus carapo* [9] and *Gymnotus omarorum* [39]. These fish have a refractory period in which they are unable to perceive electrical stimulus, called “blind period” and, they can alter their firing timing probability to avoid the refractory period, for example, *Gymnotus carapo*, when communicating, increases the probability of firing IPIs outside the range of the blind period. That is, more pulses are discharged when fish can effectively perceive electrical stimulus.

It is important to emphasize that in this paper we used a simple closed-loop stimulation to address the issue of the delay-dependent response to EOD-campled (single pulses) stimulus. Weakly electric fish are likely to integrate also information from multiple pulses and respond to stimuli in a history dependent-manner.

The approach described in this study can be expanded to more complex experiments aimed to address complex timing codes and history-dependent responses. Activity-dependent experiments are closer to natural stimulation such as in fish to fish communication because of the intrinsic bidirectional nature of the interaction. Finally, the flexibility of software-controlled real time activity-dependent closed-loop protocols open a large variety of possibilities for new experiments where the precise detection of pulses and the corresponding stimulation delivery time are crucial.
